# Targeting Seipin to Alleviate Hepatic Steatosis in Zebrafish (*Danio Rerio*)

**DOI:** 10.1002/advs.202507777

**Published:** 2025-08-13

**Authors:** Weijia Li, Yanan Shen, Zengqi Zhao, Baolin Li, Shunlang Wen, Rui Zhan, Qinghui Ai

**Affiliations:** ^1^ Key Laboratory of Aquaculture Nutrition and Feed (Ministry of Agriculture and Rural Affairs) and Key Laboratory of Mariculture (Ministry of Education) Ocean University of China 5 Yushan Road Qingdao Shandong 266003 P. R. China; ^2^ Laboratory for Marine Fisheries Science and Food Production Processes Qingdao Marine Marine Science and Technology Center Qingdao Shandong 266237 P. R. China

**Keywords:** Hepatic steatosis, Phosphatidylcholine, Seipin, Zebrafish

## Abstract

Loss of Seipin causes the absence of whole‐body adipose but abnormal liver lipid deposition in patients, and liver expression of Seipin is decreased in mice fed a high‐fat diet (HFD). However, the underlying mechanism of Seipin‐regulated liver lipid metabolism remains mysterious. Here, experiments show that over‐expression of Seipin down‐regulates HFD‐induced liver triglyceride (TG) accumulation and promotes zebrafish growth. Real‐time PCR and immunoblotting suggest that Seipin interacts with Plin2 through its second transmembrane domain to inhibit the expression of Plin2 by promoting Plin2 ubiquitination, thereby ameliorating lipid accumulation. Co‐immunoprecipitation (CoIP) experiments and Biomolecular fluorescence complementation (BiFC) analysis reveal a close interaction between Seipin and phosphatidylcholine (PC) synthesis enzyme CCTα and CHPT in zebrafish and mice. Thus, Seipin may participate in PC synthesis and increase cellular PC levels. Elevated PC levels subsequently suppress Plin2 expression. Meanwhile, CCTα, the rate‐limiting enzyme of the PC synthesis pathway, exhibited a unique regulatory role on Plin2 expression as a potential transcription factor. It is proposed that Seipin balances TG homeostasis, PC synthesis, and Plin2 expression to alleviate hepatic steatosis, providing a promising target for fatty liver disease.

## Graphical abstract

Seipin plays a crucial role in lipid metabolism. Seipin overexpression ameliorates high‐fat‐diet‐induced hepatic steatosis and enhances growth in zebrafish. Mechanistically, Seipin interacts with Plin2 to promote Plin2 degradation and enhances phosphatidylcholine synthesis via Seipin/CCTα/CHPT interactions. The elevated phosphatidylcholine levels further suppress Plin2 expression and CCTα transcriptionally regulates Plin2, revealing a Seipin‐phosphatidylcholine‐Plin2 axis in hepatic steatosis.

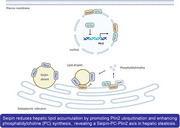

## 1 Introduction

The liver controls lipid homeostasis by ingesting, synthesizing, utilizing, storing, and exporting fatty acids (FAs). FA generates triglycerides (TGs) in the liver, and TGs stored in lipid droplets (LDs) could either enter subsequent lipid utilization pathways or become an important defense mechanism for cells against lipotoxicity.^[^ ^1^ ^]^ LDs are dynamic organelles regulating lipid storage, metabolism, and transport, and the size and number of LDs are strictly regulated. Abnormal LD accumulation is associated with many liver diseases, such as non‐alcoholic fatty liver disease (NAFLD).^[^ ^2^, ^3^ ^]^ NAFLD is a chronic liver disease characterized by the presence of LDs in more than 5% of liver cells.^[^ ^4^ ^]^ Afterward, NAFLD can progress to non‐alcoholic steatohepatitis, liver cirrhosis, liver failure, and even hepatocellular carcinoma.^[^ ^5^ ^]^ Therefore, elucidation of the potential molecular mechanism of lipid droplet accumulation during NAFLD is important for the treatment of such a disease.

LDs possess a special ultrastructure. Neutral lipids constitute the core of LDs, surrounded by a monolayer of phospholipids with numerous coating proteins.^[^ ^6^ ^]^ The stored lipids, surface phospholipids, and protein composition may play a regulatory role in the growth of LDs.^[^ ^7^, ^8^, ^9^ ^]^ Seipin is an endoplasmic reticulum (ER) transmembrane protein crucially important in LD formation. Mutations in BSCL2 encoding Seipin lead to severe lipodystrophy in patients,^[^ ^10^, ^11^ ^]^ accompanied by abnormal lipid deposition in the liver. Seipin deficiency leads to abnormal LD phenotypes. When Seipin is deleted, small and aggregated LDs or supersized LD may appear in yeast.^[^ ^12^ ^]^ The hypothesis that Seipin regulates the size of LDs revolves: (1) Seipin determines the LD formation site and effectively aggregates TGs.^[^ ^13^, ^14^, ^15^, ^16^ ^]^ (2) Seipin regulates phospholipid metabolism, especially phosphatidic acid (PA) turnover.^[^ ^17^, ^18^, ^19^, ^20^ ^]^ (3) Seipin affects ER calcium homeostasis and ER stress.^[^ ^21^, ^22^, ^23^ ^]^

However, for the surface phospholipids of LDs, phosphatidylcholine (PC) is more abundant than that of PA,^[^ ^24^, ^25^ ^]^ so it may be more effective to control the size of LD by adjusting the cellular PC level. Research has found that the knockdown of CCT1 in Drosophila S2 cells reduced the content of PC, making it easier for phosphatidylethanolamine (PE) to induce LD bending and fusion.^[^ ^26^ ^]^ Another study in C.elegans embryos has found that PC content increased after knockout of the homologue of human Seipin.^[^ ^27^ ^]^ Accordingly, Seipin may regulate LD accumulation by affecting PC content, but the underlying mechanism of Seipin regulating cellular PC levels and its potential effect on LD turnover is still unclear.

Studies have also shown that Seipin can function by interacting with a variety of proteins.^[^ ^13^, ^21^, ^28^, ^29^ ^]^ In human iPS cells, Seipin can co‐precipitate with perilipin2 (Plin2) and determine the sublocalization of Plin2.^[^ ^30^ ^]^ Plin2 is the main liver LD protein, promoting the formation of LD and protecting TAG in LDs from lipolysis.^[^ ^31^, ^32^ ^]^ The expression of Plin2 is significantly increased in fatty liver patients and high‐fat‐fed mice.^[^ ^33^, ^34^ ^]^ Thus, understanding the mechanism of Seipin regulating Plin2 through their interaction may provide a new idea for the mechanism of Seipin regulating lipid droplet formation.

Fish are the most diverse group of all vertebrates. Zebrafish is a good animal model for understanding the pathogenesis of metabolic diseases and identifying potential therapeutic targets. For example, NAFLD has been successfully simulated in zebrafish.^[^ ^35^, ^36^ ^]^ In this study, we focused on the potential mechanism of Seipin overexpression regulating liver lipid metabolism by affecting triglyceride homeostasis, phospholipid production, and lipid droplet protein in zebrafish, providing an effective therapeutic target for alleviating human fatty liver disease.

## 2 Results

### 2.1 High Fat Diet (HFD) and Oleic Acid (OA) Suppressed Seipin Expression

**Figure 1:**
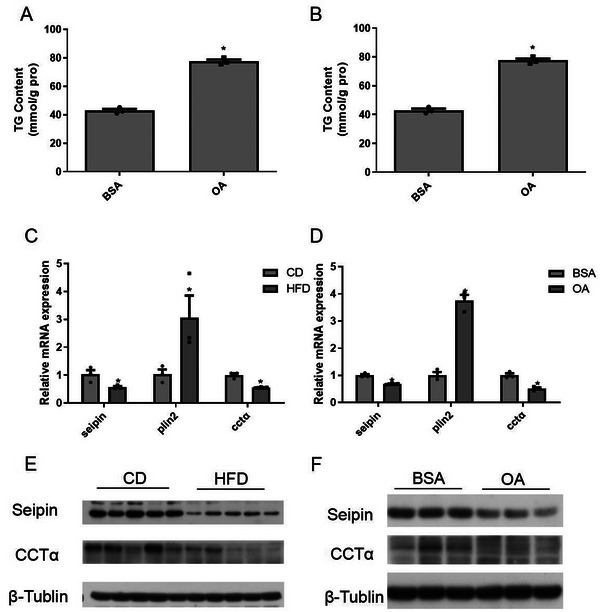
High fat diet (HFD) and oleic acid (OA) suppressed Seipin expression. AB zebrafish (n = 5) consumed an HFD or a control diet(CD) for four weeks, and ZFL cells (n = 3) were treated with 800 µm OA or BSA for 24 h. A) Liver and B) hepatocyte TG content; C) liver and D) hepatocyte *seipin* mRNA expression; E) liver and F) hepatocyte Seipin protein expression were analyzed. For statistical analysis, a one‐way analysis of variance and Tukey's test were performed. Data are presented as the means ± SEM (^*^P < 0.05).

The expression of Seipin is closely related to the lipid level. After mice consumed an HFD, the expression of Seipin in the liver was significantly down‐regulated.^[^ ^37^, ^38^ ^]^ To further explore such a phenotype in zebrafish by feeding a control diet (CD, 7% fat level) or HFD (16% fat level) and detecting the liver TG content. Compared with the CD group, along with increased serum ALT levels, elevated liver mRNA levels of proinflammatory factors (*il‐1β*, *tnfα*, *il‐6*, and *il‐8*) and accumulation of LDs in zebrafish liver (Figure A–D, Supporting Information), the liver TG content in the HFD group increased nearly two times (**Figure** **1**A). Meanwhile, we treated zebrafish liver (ZFL) cells with 800 µm OA for 24 h and found that the TG content consistently increased after the OA treatment (Figure 1B). As a consequence, the mRNA and protein expression of Seipin significantly diminished in HFD‐fed zebrafish liver and OA‐treated ZFL cells (Figure 1C–F), indicating the practicality of this model for subsequent investigation of the regulatory function of Seipin on liver lipid metabolism.

### 2.2 Seipin Overexpression Alleviated Lipid Accumulation in the Liver of Zebrafish Fed HFD

**Figure 2:**
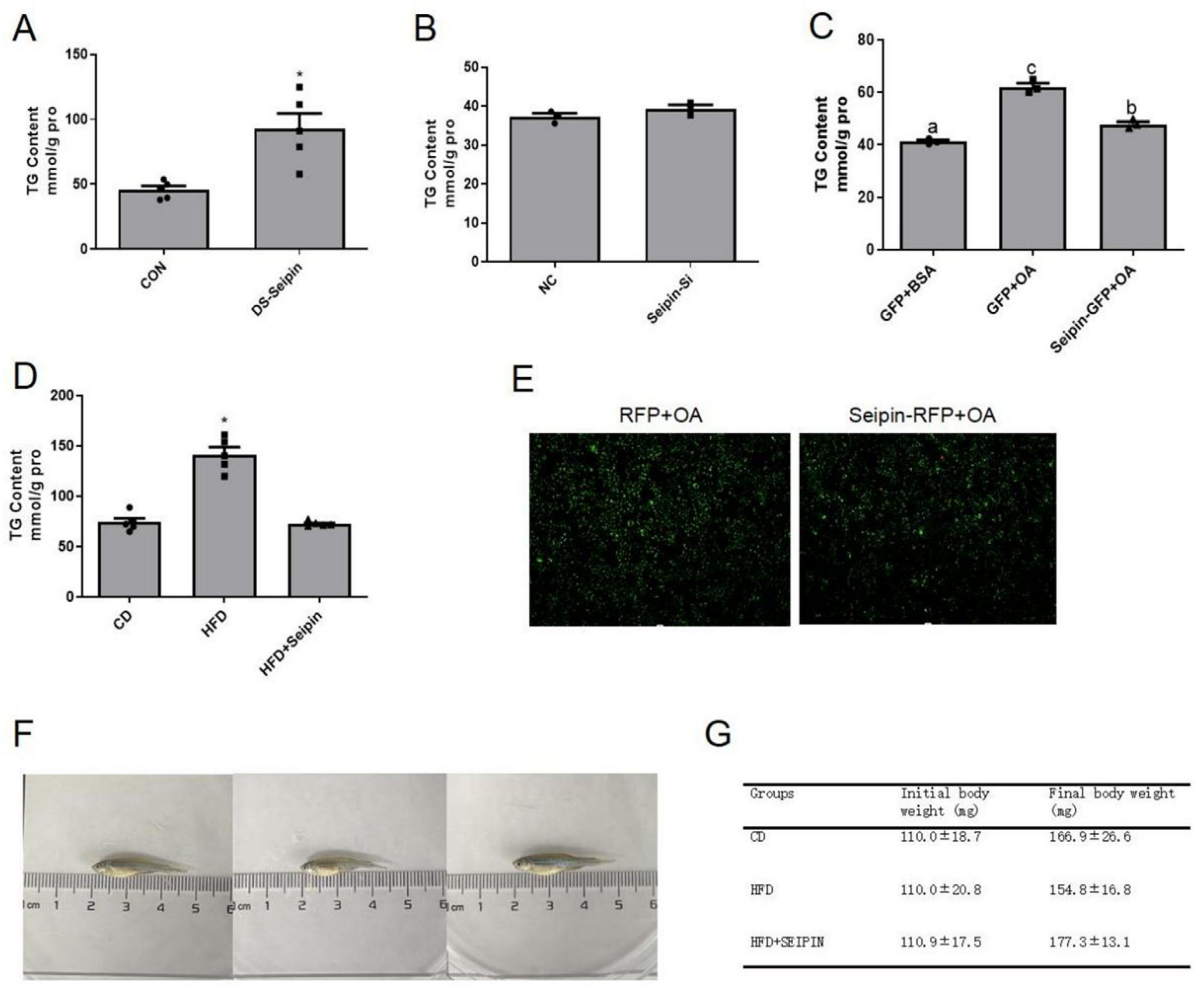
Seipin overexpression alleviated lipid accumulation in the liver of zebrafish fed HFD. A) Liver TG content of Seipin knockdown zebrafish(n = 5); B)TG content in Seipin knockdown ZFL cells (n = 3, C)TG content in Seipin over‐expressed ZFL cells (n = 3); D)liver TG content of Seipin over‐expressed zebrafish(n = 5) and (E) LD accumulation in Seipin over‐expressed ZFL cells were measured. LDs were stained with Bodipy493/503 and green fluorescence represented LDs. (F) Body weight of Seipin over‐expressed zebrafish (n = 6). For statistical analysis, Tukey's test were performed. Data are presented as the means ± SEM (^*^P < 0.05, No significance between same letters).

To understand the role of Seipin in regulating lipid storage in zebrafish, we knocked down and overexpressed Seipin in vivo and in vitro (Figure E–H, Supporting Information). We first injected Seipin‐targeted dsRNA into zebrafish to reduce Seipin expression and found that the TG content of Seipin‐interfered zebrafish liver increased significantly 36 h after injection (**Figure** **2**A), while electroporation of siRNA targeting Seipin into ZFL cells failed to change TG content 30 h after transfection (Figure 2B). In contrast, in vivo and in vitro results showed that overexpression of Seipin could reduce the increase of liver TG content and LD accumulation induced by HFD (Figure 2C–E). More importantly, accompanied by a significant reduction in liver TG accumulation, zebrafish injected with Seipin‐GFP plasmids showed an increase in body weight (Figure 2F,G), indicating that Seipin overexpression offset the negative growth effects of the HFD.

### 2.3 Seipin Affected Liver Lipid Metabolism Through Regulating Plin2

**Figure 3:**
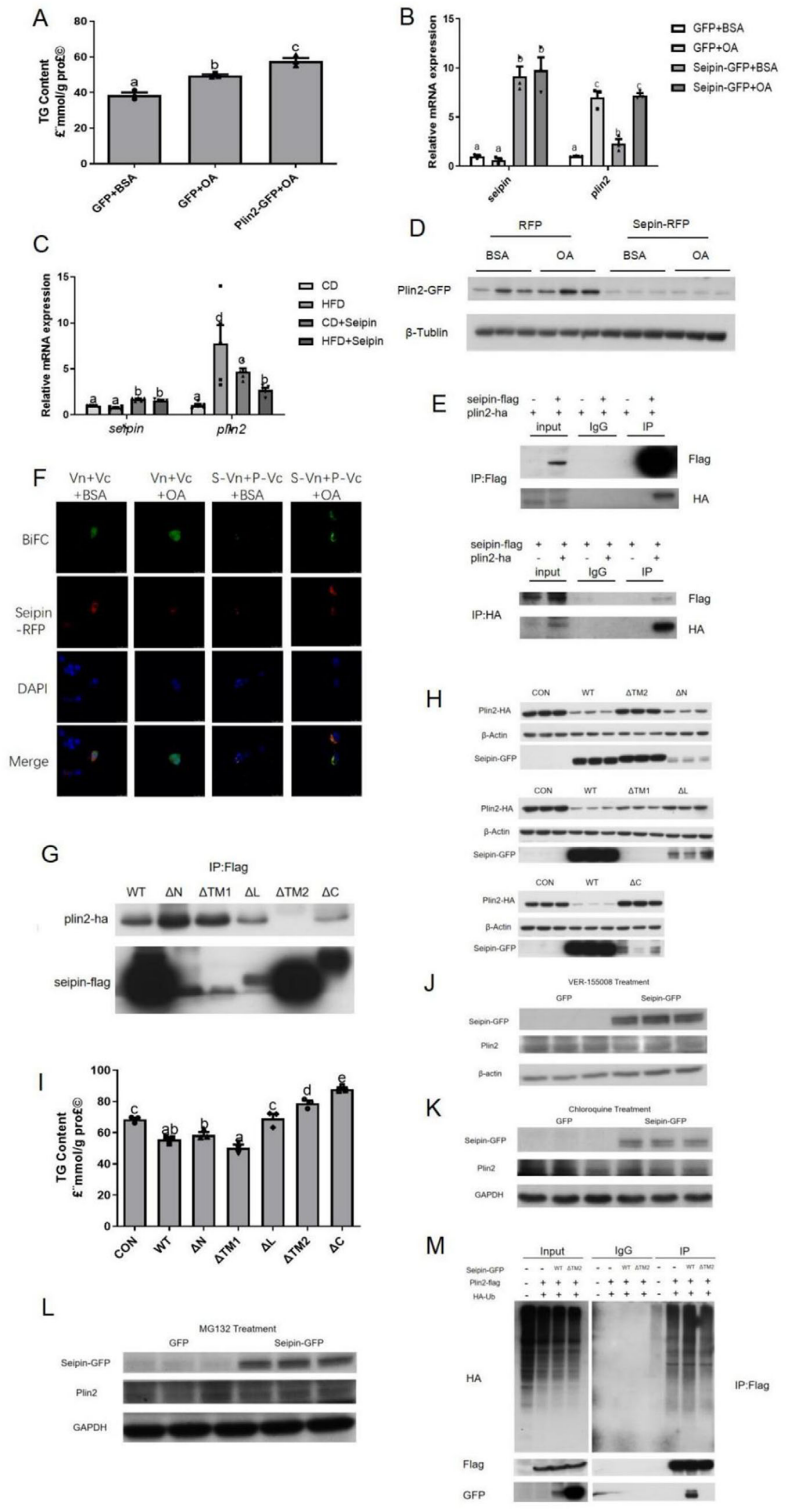
Seipin mediated Plin2 to regulate liver lipid metabolism. A) TG content in Plin2 over‐expressed ZFL cells (n = 3); B) the mRNA expression of *plin2* in Seipin over‐expressed ZFL cells (n = 3); C) liver *plin2* mRNA expression of Seipin over‐expressed zebrafish (n = 5) and D)the protein expression of Plin2‐GFP in Seipin over‐expressed ZFL cells was measured. Data are presented as the means ± SEM (No significance between same letters, *P>0.05*). E) CoIP of transfection of Seipin‐Flag and Plin2‐HA in HEK293T cells. Seipin‐Flag and Plin2‐HA were co‐immunoprecipitated by anti‐Flag or anti‐HA antibodies. F) BiFC of transfection of Seipin‐VN, Plin2‐VC, and Seipin‐RFP in HEK293T cells. The presence of GFP signal (green) signified the direct interaction between Seipin and Plin2. DAPI labeled nuclei and Seipin‐RFP (red) indicated the subcellular localization of Seipin. G) The interaction between Seipin mutants and Plin2. H) The change of Plin2 expression and I) TG content in ZFL cells after Seipin mutation. The change of Plin2 expression after J) VER‐155008, K) chloroquine, and L) MG132 treatment. M) CoIP of transfection of Plin2‐Flag, HA‐Ub, and different types of Seipin‐GFP in HEK293T cells to detect Plin2 ubiquitination. For statistical analysis, a one‐way analysis of variance was performed. Data are presented as the means ± SEM (^*^P < 0.05, No significance between same letters).

The mRNA expression of *plin2* significantly increased in the HFD and OA treatment group (Figure 1C,D). After overexpression of Plin2, the TG content of ZFL cells increased (**Figure** **3**A). When Seipin was overexpressed, both in vivo and in vitro results showed that the level of Plin2 mRNA was up‐regulated under CD and BSA treatment (Figure 3B,C). However, the *plin2* mRNA expression was down‐regulated under HFD and remained stable under OA treatment. At the same time, the level of Plin2 protein expression in ZFL cells overexpressing Seipin was significantly restrained than that in the GFP group under OA treatment. As Seipin failed to down‐regulate *plin2* mRNA expression, Seipin might regulate Plin2 protein expression through post‐translational modification (Figure 3D). Previous studies found that Seipin could modulate proteins through direct interactions between them. Since human Seipin and Plin2 could be co‐immunoprecipitated,^[^ ^30^ ^]^ we performed co‐immunoprecipitation (CoIP) experiments on zebrafish Seipin and Plin2. The results showed that HA‐labeled Plin2 could be detected in anti‐Flag immunoprecipitates, and Flag‐labeled Seipin could also be detected in anti‐HA immunoprecipitates (Figure 3E), demonstrating that there was a direct interaction between zebrafish Seipin and Plin2. Moreover, we applied biomolecular fluorescence complementation (BiFC) analysis to verify the close connection between Seipin and Plin2. The N‐terminus of GFP fused with the C‐terminus of Seipin to produce S‐VN, and the C‐terminus of GFP fused with the C‐terminus of Plin2 to produce P‐VC. The green fluorescence signal was lower in the BSA group than the OA group (Figure 3F).

To determine the regions of Seipin interacting with Plin2, we applied mutant forms of Seipin lacking either the N terminus (ΔN), first transmembrane domain (ΔTM1), ER luminal loop (ΔL), second transmembrane domain (ΔTM2), or C terminus (ΔC). TheΔTM2 mutant of Seipin lost its interaction (Figure 3G). Thus, we proposed the interaction between Seipin and Plin2 might contribute to the inhibition of Seipin on the Plin2 protein. Therefore, we overexpressed the ΔTM2 mutant of Seipin to observe its effect on Plin2 protein expression under OA treatments. Unlike the intact Seipin and ΔN, ΔTM1, and ΔL mutants of Seipin, the ΔTM2 and ΔC mutants of Seipin failed to inhibit Plin2 protein expression in ZFL cells (Figure 3H). We next overexpressed Seipin in ZFL cells and detected changes in downstream TG content. Overexpression of intact Seipin and ΔN and ΔTM1 mutants of Seipin significantly ameliorated TG accumulation after OA treatment in ZFL cells, while overexpression of the ΔL,ΔTM2, and ΔC mutants lost such effect (Figure 3I).

To detect the way of Seipin inhibiting Plin2 expression, we treated ZFL cells with Hsp70 inhibitor VER‐155008, autophagy inhibitor chloroquine, and proteasome inhibitor MG132, and found MG132 blocked the inhibitory effect of Seipin on Plin2 (Figure 3J–L). Therefore, we focused on the effect of Seipin on Plin2 ubiquitination. By co‐immunoprecipitation, we observed that Seipin enhances Plin2 ubiquitination while the ΔTM2 mutant failed to change Plin2 ubiquitination (Figure 3M). Therefore, Seipin may participate in the regulation of liver lipid accumulation by enhancing Plin2 ubiquitination through its second transmembrane domain.

### 2.4 Seipin Regulates Cellular PC Content

**Figure 4:**
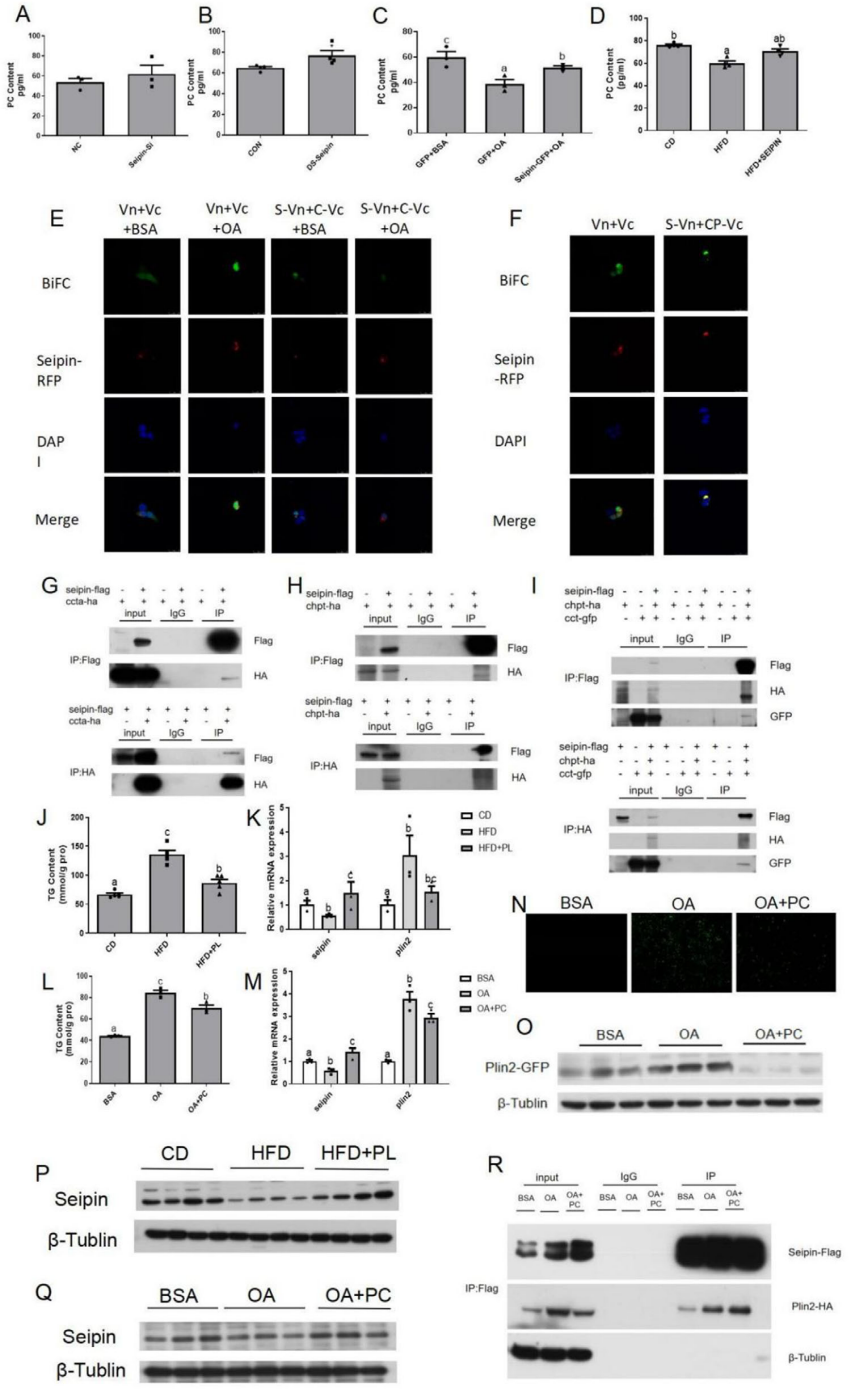
Seipin regulates cellular PC content. A) PC content in Seipin knockdown ZFL cells (n = 3); B) liver PC content of Seipin knockdown zebrafish(n = 4); C) PC content in Seipin over‐expressed ZFL cells (n = 3) and D) liver PC content of Seipin over‐expressed zebrafish(n = 4) were measured. E) BiFC of transfection of Seipin‐VN, CCTα‐VC, and Seipin‐RFP in HEK293T cells. The presence of GFP signal (green) signified the direct interaction between Seipin and CCTα. DAPI labeled nuclei and Seipin‐RFP (red) indicated the subcellular localization of Seipin. F) BiFC of transfection of Seipin‐VN, CHPT‐VC, and Seipin‐RFP in HEK293T cells. G) CoIP of transfection of Seipin‐Flag and CCTα‐HA in HEK293T cells. Seipin‐Flag and CCTα‐HA were co‐immunoprecipitated by anti‐Flag or anti‐HA antibodies. H) CoIP of transfection of Seipin‐Flag and CHPT‐HA in HEK293T cells. I) CoIP of transfection of CCTα‐GFP, Seipin‐Flag, and CHPT‐HA in HEK293T cells. CCTα‐GFP, Seipin‐Flag, and CHPT‐HA were co‐immunoprecipitated by anti‐Flag or anti‐HA antibodies. J) Liver TG content (n = 5) and K) mRNA expression of *seipin* and *plin2* (n = 3) in zebrafish consuming HFD supplemented with soybean lecithin; L)TG content (n = 3), M) mRNA expression of *seipin* and *plin2* (n = 3) and N) LD accumulation in ZFL cells treated with soybean‐lecithin‐sourced PC were analyzed. O) Western blot analysis of expression of Plin2‐GFP in ZFL cells treated with PC. P) Liver protein expression of Seipin in zebrafish consuming HFD supplemented with soybean lecithin, and Q) the protein expression of Seipin in ZFL cells treated with PC was measured. R) CoIP of transfection of Seipin‐Flag, Plin2‐HA in HEK293T cells while treating with PC. Seipin‐Flag and Plin2‐HA were co‐immunoprecipitated by anti‐Flag antibody.

Consistent with the results in *C.elegans* embryos,^[^ ^27^ ^]^ the PC content in zebrafish liver significantly increased after knocking down Seipin, while the PC content showed an upward trend without a significant difference in ZFL cells (**Figure** **4**A,B). However, when Seipin was overexpressed, the content of PC in zebrafish liver and ZFL cells also increased (Figure 4C,D). Our CoIP and BiFC results showed that Seipin interacted directly with CCTα and CHPT (Figure 4E–H). CCTα was a rate‐limiting enzyme in PC synthesis, synthesizing CDP‐choline from phosphocholine, and the downstream enzyme CHPT catalyzed CDP‐choline into PC. When we simultaneously overexpressed Seipin‐Flag, CHPT‐HA, and CCTα‐GFP in HEK293 cells, Seipin‐Flag and CCTα‐GFP could be detected in anti‐HA immunoprecipitates, and CHPT‐HA and CCTα‐GFP could be detected in anti‐Flag immunoprecipitates (Figure 4I), suggesting a three‐way interaction between Seipin, CCTα, and CHPT. Given overexpression of Seipin can increase the content of PC in cells, we fed zebrafish with HFD supplemented with soybean lecithin, the main component of which is PC. Results showed that the increase of TG content in the liver caused by HFD was significantly inhibited (Figure 4J). We next co‐incubated ZFL cells with 30 nm soybean‐lecithin‐sourced PC and 800 µm OA, and similar results were obtained. TG content was down‐regulated in the co‐incubation group (Figure 4L), and LD accumulation was reduced (Figure 4N), indicated by the weakened green fluorescence signal.

As for Plin2, the expression of Plin2 protein in ZFL cells was dramatically decreased in the co‐treatment group (Figure 4O). However, the level of Plin2 mRNA expression was also significantly down‐regulated at in vivo and in vitro levels (Figure 4K,M). Therefore, PC may exert different inhibitory effects on Plin2 expression compared with Seipin. Interestingly, the addition of PC enhanced the mRNA and protein expression of Seipin (Figure 4K,M,P,Q). At last, the interaction between Seipin and Plin2 under PC treatment was detected. PC could promote the interaction between zebrafish Seipin and Plin2 when co‐incubated with OA in HEK293 cells (Figure 4R). Under these, PC could be up‐regulated by Seipin overexpression, and PC could conversely modulate lipid accumulation by regulating the expression of Seipin and Plin2 as well as affecting their interaction.

### 2.5 Seipin Affected Liver Lipid Metabolism Through Regulating CCTα

**Figure 5:**
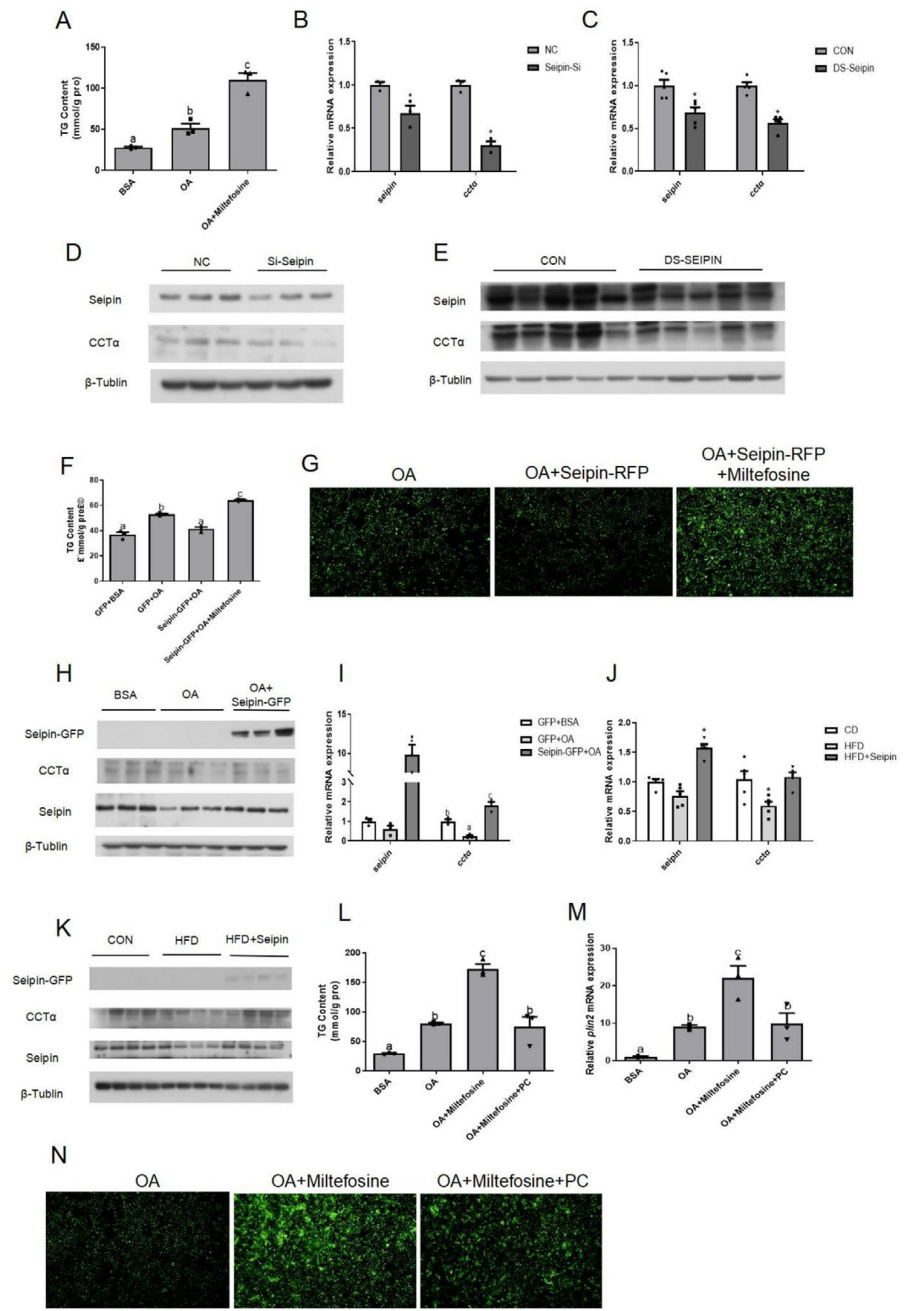
Seipin mediated CCTα to regulate lipid metabolism. 100 nm miltefosine was used to inhibit CCTα in ZFL cells. A) TG content in CCTα inhibited ZFL cells (n = 3); B) the mRNA expression of *cctα* in Seipin knockdown ZFL cells (n = 3); C) liver *cctα* mRNA expression of Seipin knockdown zebrafish (n = 5); D) the protein expression of CCTα in Seipin knockdown ZFL cells; E) liver CCTα protein expression of Seipin knockdown zebrafish; F) TG content (n = 3) and G)LD accumulationin in Seipin over‐expressed ZFL cells while inhibiting CCTα (n = 3); H)the protein and I) mRNA expression(n = 3) of CCTα in Seipin over‐expressed ZFL cells; J) liver CCTα mRNA(n = 5) and K) protein expression of Seipin over‐expressed zebrafish; L)TG content (n = 3), M) the mRNA expression of *plin2* (n = 3) and N) LD accumulationin CCTα inhibited ZFL cells while adding PC were measured. For statistical analysis, a one‐way analysis of variance and Tukey's test were performed. Data are presented as the means ± SEM (^*^P < 0.05, No significance between same letters).

Loss of CCTα could result in liver TG accumulation in mice and fish.^[^ ^39^, ^40^ ^]^ When we inhibited CCTα activity with miltefosine, the TG content of ZFL cells increased significantly (**Figure** **5**A). Due to the direct interaction between Seipin and CCTα, we hypothesized that Seipin might function by regulating CCTα. In Seipin‐interfered zebrafish liver and ZFL cells, CCTα mRNA and protein levels were significantly decreased (Figure 5B–E). Overexpression of Seipin could adversely increase CCTα mRNA and protein levels both in vivo and in vitro, revealing that Seipin exerted a substantial regulatory effect on CCTα expression (Figure 5H–K). In order to explore whether this regulatory effect played a role in liver lipid metabolism, we added 100 nm miltefosine to co‐incubate with OA in ZFL cells overexpressing Seipin. Accordingly, the co‐incubation group exhibited increased TG content and LD accumulation (Figure 5F,G), implying that overexpressed Seipin lost its mitigating effect on lipid deposition. Since CCTα is the rate‐limiting enzyme for PC synthesis, we supposed that the addition of PC could reduce the lipid accumulation induced by CCTα inhibition. As we expected, the TG content and LD accumulation of ZFL cells decreased after adding PC to the cells treated with OA and miltefosine (Figure 5L,N). Therefore, Seipin might participate in lipid metabolism by affecting CCTα and the downstream change in PC content.

### 2.6 CCTα Acted on Plin2 Expression

**Figure 6:**
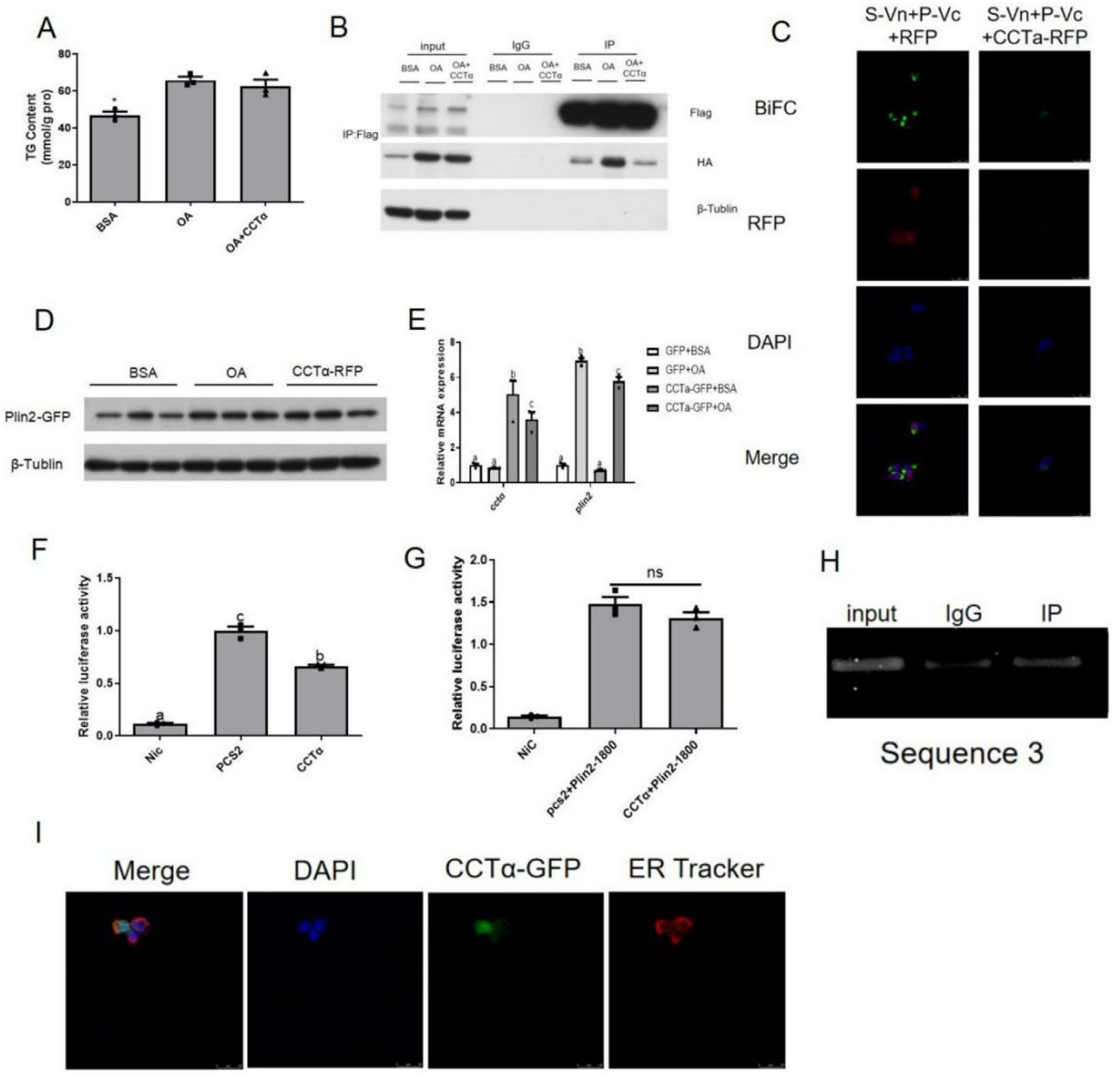
CCTα acted on Plin2 expression. A) TG content in CCTα inhibited ZFL cells (n = 3). B) CoIP of transfection of Seipin‐Flag, Plin2‐HA, and CCTα‐GFP in HEK293T cells. Seipin‐Flag and Plin2‐HA were co‐immunoprecipitated by anti‐Flag antibody. C) BiFC of transfection of Seipin‐VN, Plin2‐VC, and CCTα‐RFP in HEK293T cells. The presence of GFP signal (green) signified the direct interaction between Seipin and Plin2. DAPI labeled nuclei and red fluorescence indicated CCTα presence. D) The protein expression of Plin2‐GFP in CCTα over‐expressed ZFL cells; E) the mRNA expression of Plin2 in CCTα over‐expressed ZFL cells (n = 3); F)effect of CCTα on Plin2 promoter activity (n = 3); G) effect of CCTα on shorter term of Plin2 promoter activity (n = 3) was analyzed. H) ChIP of transfection of CCTα‐HA and Plin2 promoter. I) Cellular localization of CCTα‐GFP. For statistical analysis, a one‐way analysis of variance and Tukey's test were performed. Data are presented as the means ± SEM (^*^P < 0.05, No significance between same letters).

When we overexpressed CCTα in ZFL cells treated with OA, there was no significant difference in the TG content (**Figure** **6**A), so we speculated that other proteins might get involved in the downstream process. Since Seipin can interact with both CCTα and Plin2, the direct interaction between Seipin and CCTα might affect the regulation of Plin2 by Seipin. Therefore, we co‐overexpressed CCTα in HEK293 cells overexpressing Seipin‐Flag and Plin2‐HA and found that overexpression of CCTα greatly reduced the interaction between Seipin and Plin2 (Figure 6B). BiFC experiments also provided strong evidence when the green fluorescence signal diminished after overexpression of CCTα (Figure 6C). Nonetheless, when detecting the expression of Plin2 protein, we found that there was no significant difference in the Plin2 expression after overexpression of CCTα compared with the control group (Figure 6D), indicating that up‐regulated CCTα could not attenuate the restraint of Seipin on Plin2. Therefore, we detected the level of *plin2* mRNA expression and found that the level of *plin2* mRNA expression decreased after overexpression of CCTα (Figure 6E). Next, we cloned the Plin2 promoter sequence and performed a dual‐luciferase reporter assay experiment. The luciferase activity of Plin2‐PGL6 was reduced after overexpression of CCTα, indicating that CCTα inhibited Plin2 promoter activity (Figure 6F). Thus, CCTα might inhibit Plin2 at the transcriptional level.

To detect whether CCTα could directly modulate Plin2 mRNA expression, we performed chromatin immunoprecipitation (ChIP) to demonstrate direct binding between CCTα and the Plin2 promoter. Since the original Plin2‐PGL6 plasimid in the dual‐luciferace reporter assay experiment was constructed from 2500 bp upstream of *plin2*, we constructed a new promoter from 1800 bp upstream of *plin2* named Plin2‐1800. The luciferase activity of Plin2‐1800 was unchanged after overexpression of CCTα (Figure 6G). Therefore, we speculated that the binding area between CCTα and Plin2 promoter might locate between 1800 and 2500 bp upstream of *plin2*. To perform ChIP experiment, we divided the 700 bp into five parts and found CCTα binded to the third sequence (Figure I, Supporting Information, Figure 6H). Combined with the strong nuclear signal of CCTα (Figure 6I), CCTα might function as a transcription factor and thus directly regulates *plin2* mRNA expression.

## 3 Discussion

Seipin is closely related to human diseases, and its function and regulatory mechanisms have become a hot topic. Lack of Seipin in adipocytes and non‐adipocytes may display different results. Seipin‐deficient mouse embryonic fibroblast cells fail to differentiate into mature adipocytes, and Seipin‐deficient HepG2 cells tend to accumulate TG.^[^ ^41^, ^42^ ^]^ Therefore, how Seipin is involved in lipid metabolism remains to be explored.

Our experiments showed that Seipin deficiency led to an increase in TG content in zebrafish liver, consistent with a previous study that Seipin regulated liver lipid deposition through SCD1 in mouse liver^[^ ^38^ ^]^ indicating that Seipin plays an important role in liver lipid metabolism. The HFD reduced Seipin expression, and Seipin overexpression resulted in a decrease in TG levels in the liver of zebrafish fed with the HFD, consistent with the results of seipin mediating hepatic lipid metabolism through regulating calcium homeostasis and ER stress in mice,^[^ ^37^ ^]^ suggesting that Seipin may become a valuable target for alleviating high‐fat‐induced liver lipid deposition. More importantly, overexpression of Seipin increased the body weight of zebrafish and ameliorated the growth inhibition effect of an HFD while reducing the TG level in the liver of HFD zebrafish to a similar level of the CD group. Due to the shortage of fish meal resources, the wide use of high‐fat feed has become an important alternative in the aquaculture industry. However, it leads to liver fat deposition in cultured fish, inhibits fish growth and survival, and damages its economic value.^[^ ^43^ ^]^ Therefore, targeting Seipin might also help to improve the utilization of fat in feed and promote the healthy development of aquaculture.

Seipin can function through interacting with a variety of proteins. Generally, Seipin interacts with LDAF1 to aggregate TG,^[^ ^13^ ^]^ interacts with metabolic enzymes such as AGPAT2, lipin‐1, and GPATs to affect PA metabolism^[^ ^28^, ^29^ ^]^ interacts with the ER calcium pump SERCA to affect cell calcium homeostasis.^[^ ^21^ ^]^ Previous studies have also suggested a direct interaction between Seipin and Plin2, and the close connection can affect the localization of Plin2 to LDs.^[^ ^30^ ^]^ In this study, we focused on the effect of Seipin on the expression and function of Plin2, and we believed that the interaction between Seipin and Plin2 is the key to the regulation of cellular lipid metabolism. Plin2 is a typical characteristic protein in fatty liver disease, and its overexpression is related to LD aggregation. Even in serum‐free cultured cells, overexpression of Plin2 can increase TG content.^[^ ^44^ ^]^ Our experiments also found that plin2 overexpression increased TG content in ZFL cells after OA incubation, while the introduction of Seipin suppressed such an impact. Plin2 could be degraded through chaperone‐mediated autophagy (CMA) and ubiquitin/proteasome pathways. Plin2 associated with LDs was reported to be degraded through CMA,^[^ ^45^ ^]^ while Plin2 not associated with LDs was degraded through its ubiquitination.^[^ ^46^ ^]^ When we overexpressed Seipin, the localization of Plin2 changed from aggregation to dispersion distribution, and its localization was consistent with Seipin (Figure J, Supporting Information). Seipin was mainly localized on ER membrane, the interaction between Seipin and Plin2 might make Plin2 distributed on the ER rather than surround LDs, thus promote Plin2 ubiquitination and LDs degradation. Therefore, the cooperation between Seipin and Plin2 may play an important role in Seipin ’s regulation of liver lipid metabolism.

Later, we found for the first time that Seipin can interact with two enzymes in the PC de novo synthesis pathway, that is CCTα and CHPT. Due to the three‐way interaction, the CDP‐choline catalyzed by CCTα might be more associated with CHPT, promoting PC synthesis. We also confirmed the three‐way interaction of mouse Seipin, CCTα, and CHPT in HEK293T cells (Figure K, Supporting Information). Based on the function of Seipin in promoting PC synthesis, the exogenous addition of PC can also reduce liver fat deposition caused by lipid overload. Because of its cylinder shape, PC is beneficial to cover the surface of LDs.^[^ ^47^ ^]^ When the PC / PE ratio is reduced, the interaction between the internal lipid and the surface protein is increased, making it easier to bind the proteins, such as Plin2.^[^ ^48^ ^]^ When the level of PC is elevated, Plin2 may be difficult to bind to LDs, and the interaction between Plin2 and Seipin increases, thus facilitating Plin2 degradation. We also detected an increased Seipin/Plin2 interaction after PC under BSA treatment (Figure L, Supporting Information). Therefore, overexpression of Seipin may also inhibit Plin2 by increasing PC content.

Interestingly, when we exogenously added PC, the expression of Seipin increased, so there was a mutually reinforcing relationship between Seipin expression and cellular PC content, and it might be the reason for the increase of endogenous Seipin when we exogenously overexpressed Seipin‐GFP. Overexpression of Seipin increased PC content, in turn promoting Seipin expression. Therefore, we doubled the concentration of Seipin plasmids used for electroporation. Results showed that high levels of Seipin overexpression lost its lipid‐lowering effect and, reversely increased cellular TG content and LD accumulation (Figure M–O, Supporting Information). Therefore, mild overexpression of Seipin is beneficial to liver health, while excessive overexpression of Seipin might lead to cell homeostasis disequilibrium and become detrimental.

CCTα is the rate‐limiting enzyme for PC synthesis.^[^ ^49^ ^]^ When cells were treated with CCTα activity inhibitors, the PC content of the cells decreases (Figure P, Supporting Information), and the TG content increases, consistent with CCTα knockdown in mice and fish.^[^ ^39^, ^40^ ^]^ The reduction of PC on the surface of the LDs, facilitates the coalescence of LDs and the localization of proteins that promote LD formation, such as Plin2, to LDs.^[^ ^9^ ^]^ Cytosolic Plin2 inclines to degradation than Plin2 binding to LDs.^[^ ^45^ ^]^ Indeed, our experiments found that the expression of the Plin2 protein was significantly increased after inhibition of CCTα. However, CCTα showed an unusual regulatory role in Plin2 expression. Though CCTα overexpression down‐regulated Plin2 promoter activity, PC addition failed to play a similar role (Figure Q, Supporting Information). Combined with its direct interaction with Plin2 promoter, CCTα may act beyond its traditional function as a transcription factor.

In the relationship between CCTα and Plin2, we found that CCTα regulated Plin2 through two distinct mechanisms, including transcriptional repression of *plin2* and interference with Seipin/Plin2 interactions. Since the Seipin/Plin2 interaction was crucial for Plin2 ubiquitination, CCTα might disrupt Seipin‐dependent Plin2 degradation by inhibiting Seipin/Plin2 interactions. In this case, overexpression of CCTα simultaneously suppressed Plin2 transcription and degradation, which led to constant Plin2 protein levels. Therefore, although CCTα was up‐regulated after Seipin overexpression and Plin2 was modulated by CCTα through different mechanisms, Plin2 regulation by Seipin overexpression was independent of CCTα.

In the BiFC experiment of Seipin interacting with CCTα, our results showed that when cells were incubated wih BSA, the interaction between Seipin and CCTα was present in the cytoplasm and nucleus, while when cells were incubated with OA, the co‐localization of the two proteins appeared only in the nucleus. Due to the cytoplasmic localization of CHPT, we speculated that the cytoplasmic interaction between Seipin and CCTα may be the key to promoting PC synthesis. The decrease in the interaction between Seipin and CCTα in the cytoplasm during OA incubation might prove that the PC synthesis was restrained. However, the physiological significance of the interaction between Seipin and CCTα in the nucleus remained unclear.

Regarding the function of each domain of Seipin, we found that after mutating the second transmembrane domain, overexpression of Seipin no longer inhibited Plin2 expression and TG accumulation. Since Seipin interacted with another LD protein FIT2 through the second transmembrane domain (Figure R, Supporting Information), the second transmembrane domain of zebrafish Seipin might be fundamental in regulating the binding of Seipin to LD protein. When the ER luminal loop was mutated, the function of overexpression of Seipin in inhibiting TG accumulation is also weakened. We speculate that the reason may be the increased level of PA. It has been found that mammalian Seipin interacts with lipin1 and agpat2 through the ER luminal loop to accelerate the turnover of PA. When the C terminus was mutated, overexpression of Seipin increased Plin2 expression and aggravated TG accumulation in ZFL cells. Because of the tight interaction of Seipin, Plin2, and cellular PC level, we detected the change of PC content after mutation of Seipin, and found that overexpression of Seipin failed to increase the content of PC in cells when only the C terminus was mutated (Figure S, Supporting Information). Therefore, the C terminus of zebrafish Seipin might play a key role in PC metabolism.

**Figure 7:**
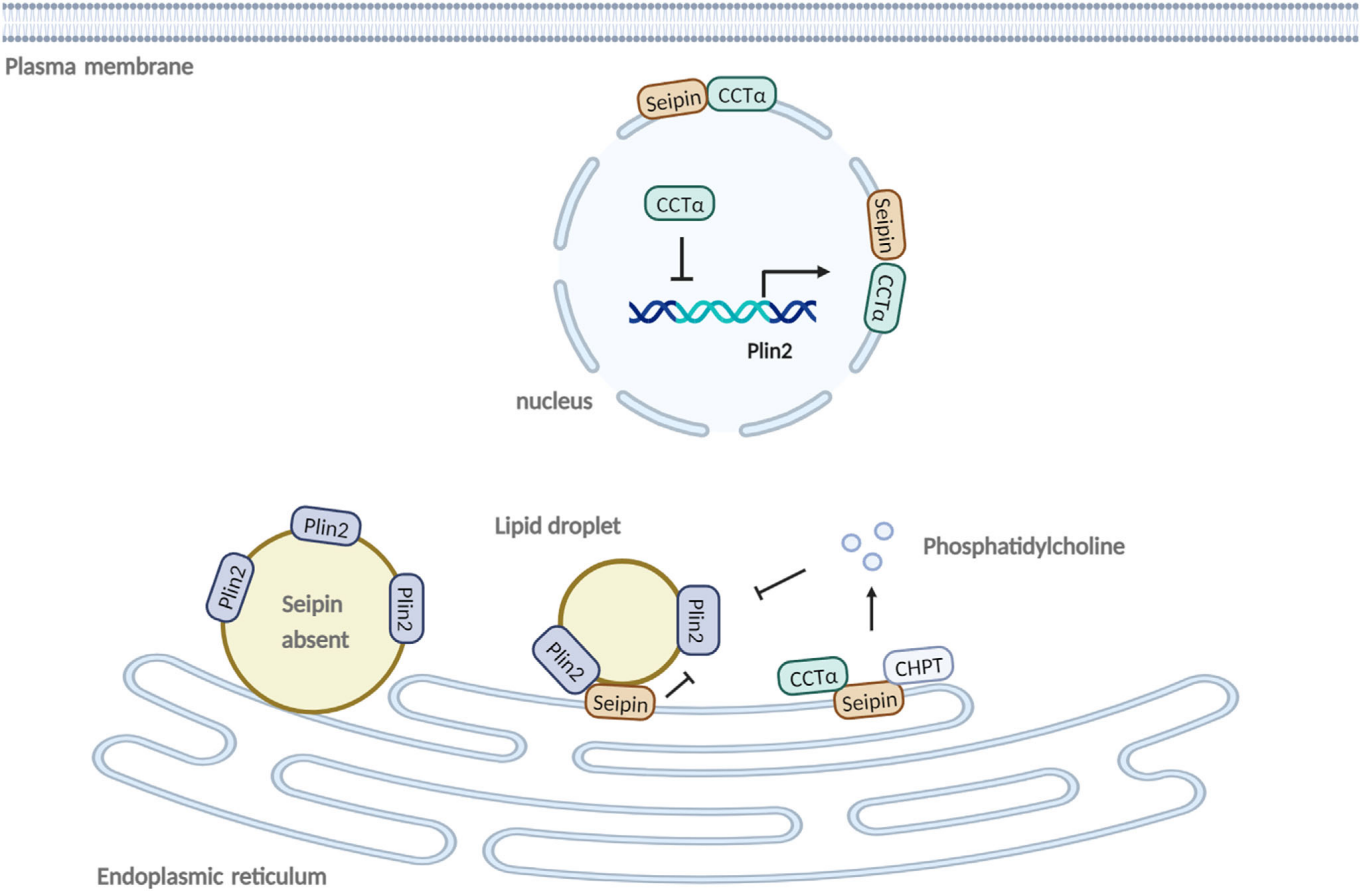
A working model linking Seipin and LD accumulation, PC synthesis, and Plin2 expression in zebrafish (*Danio rerio*). Seipin interacts with Plin2 and down‐regulates Plin2 expression, interacts with CCTα and CHPT to promote PC synthesis, and increases cellular PC levels, inhibit Plin2 expression, thus alleviating liver TG and LD accumulation.

Finally, a previous study has demonstrated Seipin overexpression alleviated hepatic TG accumulation through the calcium signaling pathway in mice.^[^ ^37^ ^]^ While our study focused on TG homeostasis, PC synthesis, and Plin2 expression after Seipin overexpression in zebrafish, we recognized that calcium signaling might also play a role in Seipin‐mediated liver lipid metabolism. Therefore, we detected ionomycin‐induced calcium release after Seipin overexpression and found reduced calcium flux in the Seipin overexpression group (Figure T, Supporting Information), suggesting that Seipin might play a regulatory role in cellular calcium homeostasis. Considering that ionomycin triggered calcium release from most intracellular stores, and mitochondrial calcium levels were notably diminished in Seipin‐overexpressing HepG2 cells^[^ ^37^ ^]^ the decreased calcium release might reflect altered mitochondrial calcium handling. Given the critical role of mitochondrial calcium homeostasis in maintaining mitochondrial function, future studies are needed to specifically investigate the mechanistic relationship between Seipin overexpression and calcium dynamics in zebrafish liver.

In summary, the significant effect of Seipin overexpression on downregulating liver lipid accumulation in zebrafish indicates that it can be a promising target for alleviating hepatic steatosis (**Figure** **7**).

## 4 Conclusion

Our study demonstrated that Seipin can balance liver lipid metabolism by regulating TG homeostasis, PC synthesis, and LD‐related protein expression, thereby becoming an effective target for alleviating fatty liver disease.

## 5 Experimental Section

### Animal Experiments

All animal experiments in this study were carried out in strict accordance with the ‘ Laboratory Animal Management Regulations ’ (Chinese Order No. 676 of the State Council, revised on March 1, 2017).

The zebrafish used in this experiment were provided by the Chinese National Zebrafish Resource Center (Wuhan, China). The first feeding experiment was to evaluate the effect of a high‐fat diet on zebrafish and the function of PC. 81 experimental fish (two months old) were randomly divided into three groups (three tanks in each group, nine fish per tank) and fed with a control diet (CD, 7% fat), high‐fat diet (HFD, 16% fat) and HFD supplemented with soybean lecithin diet, according to Table S1. During the feeding period, the light‐dark cycle was 12/12 h, the water temperature was maintained at 28 °C, and the experimental fish were fed twice a day for four weeks. In the second experiment, Seipin and fed CD were overexpressed and HFD. 28 experimental fish (two months old) were divided into four groups (seven in each group) and injected with GFP or Seipin‐GFP plasmid at a dose of 2 µg / (g b.w) every other day. The experiment lasted for seven days, and the experimental fish were fed twice a day. In the third experiment, 20 zebrafish (two months old) were divided into two groups (ten in each group). Zebrafish Seipin‐targeted dsRNA at a dose of 2 µg / (g b.w) was injected intraperitoneally, or the same amount of dsRNA‐control was cultured for 36 h. Finally, the experimental fish were weighed after anesthesia with MS222 and liver samples were obtained for further analysis.

### Cell Culture and Treatment

The ZFL cell line was obtained from the Chinese Academy of Agricultural Sciences (Beijing, China). The cells were cultured in DMEM / F12 medium (Vivo Cell, China) containing 10% FBS (ExCell, China) and 1% antibiotics (Solarbio, China) at 28 °C and 5% CO2. ZFL cells were treated with 800 µm OA or BSA in 6‐well or 12‐well plates at a density of 1.0 × 10^6^ cells mL^−1^ to explore the response of ZFL cells to OA. PC (Selleck, USA) was added to 800 µOA to explore the role of PC under high‐fat conditions. Miltefosine (MCE, USA) was added to explore the effect of CCTα on liver lipid metabolism. BSA and OA in the control group were added with the same amount of DMSO. To explore the role of Seipin in ZFL cell response to OA, Seipin‐siRNA and Seipin‐GFP plasmid were electroporated into ZFL cells to knockdown and overexpression of Seipin, respectively. After treatment, cells were lysed in the well and collected for subsequent analysis.

### RNA Extraction and RT‐PCR

According to the instructions, total RNA was extracted with a Trizol reagent (Vazyme, China). The quality of RNA was detected by 1.2% denaturing agarose gel electrophoresis, and cDNA synthesis was performed using Hiscript III RT Supermix for qPCR Kit (Vazyme, China) according to the instructions. The mRNA level was measured using a quantitative thermal cycler (Bio‐rad, USA) applying a ChamQ Universal SYBR qPCR Master Mix (Vazyme, China). The level of each gene was normalized to the level of β‐actin using the 2‐^ΔΔct^ method.^[^ ^50^ ^]^

### Western Blot

Total protein was extracted from zebrafish liver and ZFL cells using RIPA lysate containing protease inhibitors and phosphatase inhibitors. The BCA Protein Assay Kit (Beyotime, China) was used to determine the protein concentration according to the instructions. Western blot analysis was carried out according to the previous method.^[^ ^51^ ^]^ The main specific antibody used in this study was anti‐Seipin (ab181761, Abcam, USA), anti‐CCTα (YN2909, ImmunoWay, USA), anti‐GFP (sc9996, Santa Cruz, USA), anti‐Flag (14793S, CST, USA), anti‐HA (3724S, CST, USA) and anti‐β‐Tublin (AF7011, Affinity, USA). The fluorescence detection was developed with Beyo ECL Plus Reagent (Beyotime, China) and scanned using an Epson Perfection V33 scanner (China) according to the manufacturer's instructions.

### CoIP

The CoIP experiment was performed according to the previous method.^[^ ^52^ ^]^ The plasmids used in the experiment were constructed into PCS2+ vectors to create Seipin‐Flag, Plin2‐HA, CCTα‐HA, and CHPT‐HA, and were harvested 36 h after transfection into HEK293T cells. Cells were then lysed with Cell lysis buffer for Western and IP (Beyotime, China), and the supernatant was collected for subsequent experiments. The supernatant was incubated with Pierce ANTI‐HA agarose (Thermo Fisher Scientific, USA), ANTI‐FLAG M2 Affinity Gel (Sigma, USA), and IgG beads (Sigma, USA). Proteins were then decrosslinked from agarose beads using HA peptides (MedChem Express, USA) or Flag peptides (MedChem Express, USA) for further analysis.

### BiFC

Zebrafish Seipin was cloned into the VN173 vector (HonorGene, China), and Plin2, CCTα and CHPT were cloned into the VC155 vector (HonorGene, China). The plasmid was transfected into HEK293T cells by lipo2000 (Invitrogen, USA). HEK293T cells were cultured in DMEM medium containing 10% FBS and 1% antibiotics, and cultured in 37 °C, 5% CO2 incubator. After 36 h, fixation and staining were performed, and a laser scanning confocal microscope was observed. Nuclear staining with DAPI was purchased from Beyotime, China.

### Subcellular Colocalization Analysis

Seipin‐RFP and Plin2‐GFP were electroporated into ZFL cells to detect Plin2 subcellular localization after Seipin overexpression. After 36 h, fixation and staining were performed, and a laser scanning confocal microscope was observed. ZFL cells were treated with 800 µm OA for 24 h before fixation. Subcellular colocalization between RFP and GFP was analyzed under Image J software.

### Double Fluorescent Reporter Enzyme Experiment

The reporter plasmid pGL6‐plin2 and the expression plasmid pCS2‐CCTα were constructed by homologous recombination. HEK293T cells were transfected with 200 ng pGL6 basic plasmid or pGL6‐plin2 promoter plasmid, 600 ng pCS2 plasmid or pCS2‐CCTα plasmid, and 20 ng CMV plasmid using Lipo2000. Cells were collected 24 h after transfection, and luciferase activity was detected using a dual luciferase reporter assay system (Transgenic Biotech, China).

### Measurement of AST, ALT, TG, and PC content

The AST, ALT and TG content was determined by the kit (Applygen, China), and the PC content was determined by the ELISA kit (Jingmei, China), all according to the kit instructions.

### Calcium Flux Analysis

HEK293T cells were transfected with Seipin‐GFP and pGP‐CMV‐jRGECO1a and incubated for 36 h. Calcium release was stimulated with 5 µm ionomycin (Beyotime, China). The calcium signal was detected by a multimode microplate reader (BioTek, USA) and presented as the mean of the relative change in fluorescence intensity divided by baseline intensity(ΔF/F).

### Data Analysis

All data were expressed as mean ± standard error (mean ± SEM). All statistical analyses were performed using Statistical Product and Service Solution 19.0 (SPSS, USA). Student's *t*‐test was used to compare the means between the two groups, and a one‐way analysis of variance was used for multiple groups. *P < 0.05* was considered statistically significant.

## Conflict of Interest

The authors declare no conflict of interest.

## Author Contributions

W. L., Y. S., B. L., and S. W. performed experiments. R. Z. reared the experimental fish. Q. A., W. L., Y. S., and Z. Z. conceptualized and designed the research. All authors participated in data analysis. W. L. drafted, and all other authors revised the manuscript.

## Supporting information



Supporting Information

## Data Availability

The data that support the findings of this study are available from the corresponding author upon reasonable request.
