# Supplementary material for: Targeting Seipin to Alleviate Hepatic Steatosis in Zebrafish (Danio Rerio)
Source: Adv Sci (Weinh). 2025 Aug 13;12(37):e07777. doi: 10.1002/advs.202507777 (PMC12499419; doi:10.1002/advs.202507777)
Supplement: Supplementary file 1 — Supporting Information [file ADVS-12-e07777-s001.docx]

**Supporting Information**

**Table S1 Formulation of the experimental diet for zebrafish**

| Ingredients^1^ | Diets | | |
| --- | --- | --- | --- |
|  | CD | HFD | HFD+PL |
| Casein | 324.42 | 324.42 | 324.42 |
| Gelatin | 50.00 | 50.00 | 50.00 |
| Fish oil | 70.00 | 160.00 | 80.00 |
| Starch | 280.00 | 280.00 | 280.00 |
| Cellulose | 210.58 | 120.58 | 120.58 |
| Monocalcium phosphate | 10.00 | 10.00 | 10.00 |
| Vitamin premix | 10.00 | 10.00 | 10.00 |
| Mineral premix | 40.00 | 40.00 | 40.00 |
| Choline chloride | 5.00 | 5.00 | 5.00 |
| Soybean Lecithin | 0.00 | 0.00 | 80 |
| Total | 1000.00 | 1000.00 | 1000.00 |

^1^All ingredients were obtained from Great Seven Biotechnology Co., Ltd., Shandong, China.

**Supplementary figure**

**
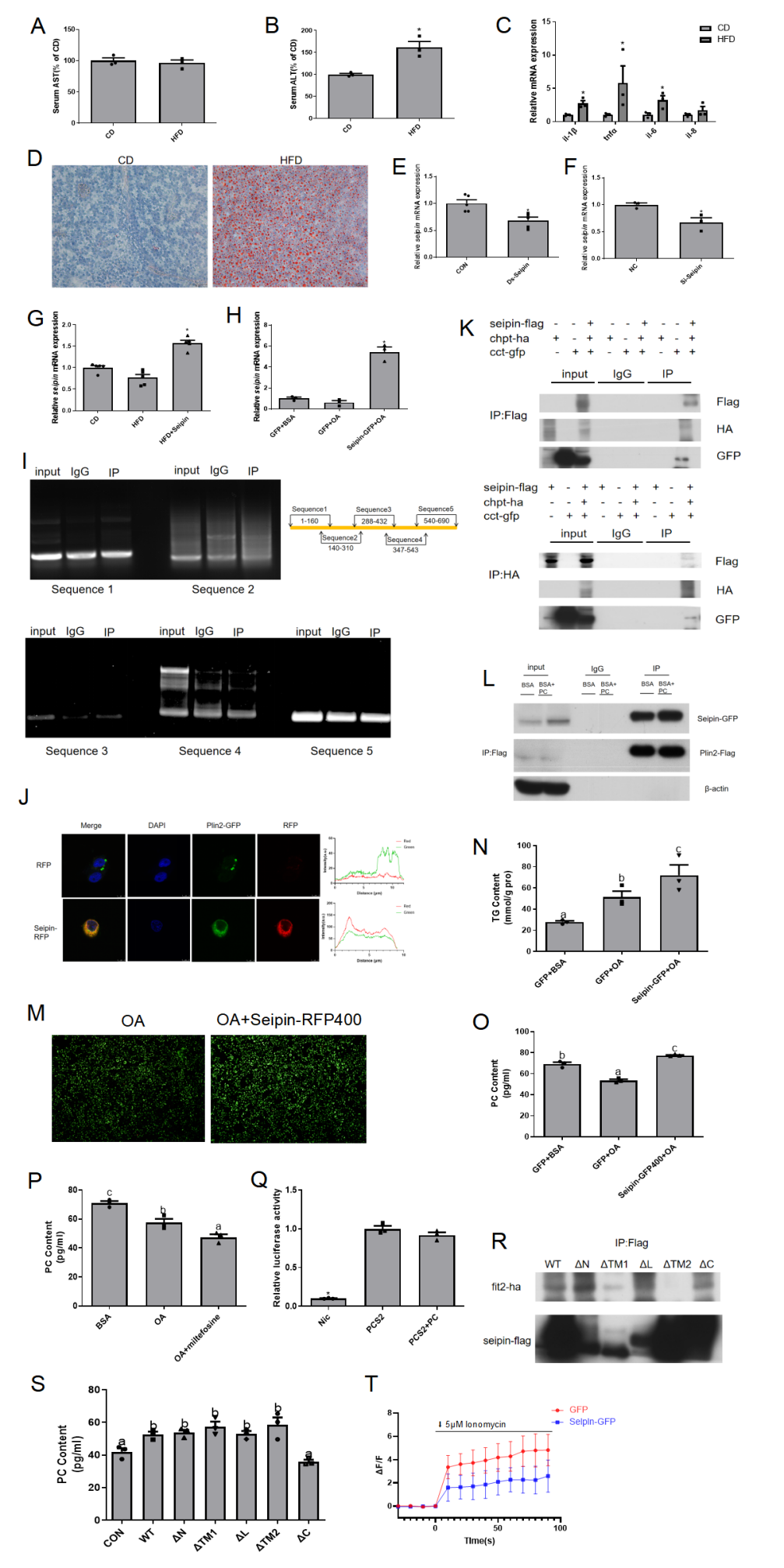
**

**Supplementary figure.** (A) AST and (B) ALT levels in zebrafish serum fed with HFD (serum from five fish in one sample, n=3), and (C) mRNA levels of proinflammatory factors (*il-1β*, *tnfα*, *il-6* and *il-8*) in liver were detected (n=3). (D) Liver oil red staining of zebrafish fed with HFD. MRNA expression of *seipin* after *seipin* knockdown (E) *in vivo* (n=5) and (F) *in vitro* (n=3); mRNA expression of *seipin* after Seipin-GFP overexpression (G) *in vivo* (n=5) and (H) *in vitro* (n=3) were analyzed. (I) Interaction between CCTα-HA with different regions of Plin2 promoter. (J) Cellular localization of Plin2-GFP after tansfected with Seipin-RFP. (K) CoIP of transfection of mouse CCTα-GFP, Seipin-Flag, CHPT-HA in HEK293T cells. CCTα-GFP, Seipin-Flag and CHPT-HA were co-immunoprecipitated by anti-Flag or anti-HA antibodies. (L) Seipin/Plin2 interaction after addition of PC under BSA treatment. (M) LD accumulation, (N) TG content (n=3), and (O) PC content (n=3) of ZFL cells electroporated with high concentration of Seipin-GFP plasmids were measured. (P) PC content of miltefosine treated ZFL cells (n=3) was analyzed. (Q) Plin2 promoter activity after PC treatment. (R)The interaction between Seipin mutant and FIT2. (S) The change of PC content in cells after Seipin mutation. (T) Calcium flux changes induced by 5 μM ionomycin after Seipin overexpression (n=3). For statistical analysis, a one-way analysis of variance and Tukey's test were performed. Data are presented as the means ± SEM (**P < 0.05*, No significance between same letters).
